# Genetic Diversity, Antimicrobial Susceptibility, and Biofilm Formation of *Cronobacter* spp. Recovered from Spices and Cereals

**DOI:** 10.3389/fmicb.2017.02567

**Published:** 2017-12-19

**Authors:** Yuanhong Li, Huan Yu, Hua Jiang, Yang Jiao, Yaodong Zhang, Jihong Shao

**Affiliations:** ^1^School of Public Health, Xuzhou Medical University, Xuzhou, China; ^2^Department of Pharmacy, Wuhan No.1 Hospital, Wuhan, China

**Keywords:** *Cronobacter* spp., Multilocus sequence typing, serotyping, antimicrobial susceptibility, biofilm formation

## Abstract

*Cronobacter* species are important food-borne opportunistic pathogens which have been implicated in the cause of necrotizing enterocolitis, sepsis, and meningitis in neonates and infants. However, these bacteria are routinely found in foodstuffs, clinical specimens, and environmental samples. This study investigated the genetic diversity, antimicrobial susceptibility, and biofilm formation of *Cronobacter* isolates (*n* = 40) recovered from spices and cereals in China during 2014–2015. Based on the *fus*A sequencing analysis, we found that the majority (23/40, 57.5%) of *Cronobacter* isolates in spices and cereals were *C. sakazakii*, while the remaining strains were *C. dublinensis* (6/40, 15.0%), *C. malonaticus* (5/40, 12.5%), *C. turicensis* (4/40, 10.0%), and *C. universalis* (2/40, 5.0%). Multilocus sequence typing (MLST) analysis produced 30 sequence types (STs) among the 40 *Cronobacter* isolates, with 5 STs (ST4, ST13, ST50, ST129, and ST158) related to neonatal meningitis. The pattern of the overall ST distribution was diverse; in particular, it was revealed that ST148 was the predominant ST, presenting 12.5% within the whole population. MLST assigned 12 isolates to 7 different clonal complexes (CCs), 4, 13, 16, 17, 72, 129, and 143, respectively. The results of O-antigen serotyping indicated that *C. sakazakii* serotype O1 and O2 were the most two prevalent serotypes. The antimicrobial susceptibility testing showed that the 40 *Cronobacter* isolates were susceptible to most of the antibiotics tested except for ceftriaxone, meropenem, and aztreona. Of the 40 *Cronobacter* strains tested, 13 (32.5%) were assessed as weak bioflim producers, one (2.5%) was a moderate biofilm producer, one (2.5%) was strong biofilm producer, and the others (62.5%) were non-biofilm producers. MLST and O-antigen serotyping have indicated that *Cronobacter* strains recovered from spices and cereals were genetically diverse. Isolates of clinical origin, particularly the *C. sakazakii* ST4 neonatal meningitic pathovar, have been identified from spices and cereals. Moreover, antimicrobial resistance of *Cronobacter* strains was observed, which may imply a potential public health risk. Therefore, the surveillance of *Cronobacter* spp. in spices and cereals should be strengthened to improve epidemiological understandings of *Cronobacter* infections.

## Introduction

The *Cronobacter* genus, belonging to the family Enterobacteriaceae, includes seven species: *C. sakazakii, C. malonaticus, C. dublinensis, C. muytjensii, C. turicensis, C. universalis*, and *C. condimenti* (Iversen et al., 2008; Joseph et al., 2012). Among them, three species in the genus *Cronobacter*, including *C. sakazakii, C. malonaticus*, and *C. turicensis* have been implicated in fatal neonatal infections resulting in sepsis, necrotizing enterocolitis and meningitis, with a high mortality rate and probability of neurological sequelae (Hunter and Bean, 2013; Ogrodzki and Forsythe, 2015). Although neonatal infections caused by *Cronobacter* spp. were highlighted, recent studies indicated that these bacteria can cause illness in both infants and adults, especially for newborns, the elderly, and individuals with weakened immune systems (Patrick et al., 2014; Alsonosi et al., 2015). Outbreaks of *Cronobacter* infections have been reported in many countries in recent years (Friedemann, 2009; Holý et al., 2014; Patrick et al., 2014).

The genus *Cronobacter* includes many ubiquitous species that are found in foodstuffs or raw materials, and clinical specimens as well as environmental samples (Reich et al., 2010; Alsonosi et al., 2015; Song et al., 2016; Brandão et al., 2017), but the exact reservoir and routes of transmission has still not been ascertained (Sani and Odeyemi, 2015). Understanding the transmission routes (e.g., waterborne, foodborne, or environmental) and vehicles (e.g., powdered infant formula, vegetables, meat, spices, or cereals) of a *Cronobacter* outbreak is of great public health importance. Thus, evaluation of a wide variety of foods might be necessary to reveal possible routes for transmission of infections caused by the genus *Cronobacter*.

Molecular typing techniques have become an important tool to study the genetic diversity of *Cronobacter* spp. and to trace individual strains that cause human infections. In recent years, a number of molecular typing techniques such as MLST (Baldwin et al., 2009), PCR-restriction fragment length polymorphism (PCR-RFLP) (Vlach et al., 2017), pulsed field gel electrophoresis (PFGE) (Lou et al., 2014), amplified fragment length polymorphism (AFLP) (Turcovský et al., 2011), and random amplified polymorphic DNA (RAPD) (Drudy et al., 2006), have been established to differentiate these pathogens. Among these typing techniques, MLST is currently considered to be the best tool for epidemiological studies of *Cronobacter* spp. due to its high reproducibility and discriminatory ability. Serotyping is another important diagnosis tool widely used for identifying food-borne pathogens. Recent studies indicated that *Cronobacter* spp. have been differentiated into 17 serotypes by PCR-based O-antigen serotyping assays targeting the *wzx* (O-antigen flippase) and the *wzy* (O-antigen polymerase) genes (Jarvis et al., 2011, 2013; Sun et al., 2011, 2012a,b). The development of these molecular techniques is greatly helpful to distinguish *Cronobacter* species and may further assist in epidemiological investigation of outbreaks of *Cronobacter* infections.

Owing to the improper and abusive usage of antimicrobial agents, the emergence and spread of multidrug-resistant strains have become a serious threat to public health worldwide. Current studies indicated that *Cronobacter* spp. seemed to be less resistance to commonly used antibiotics compared to other foodborne pathogens such as *Listeria monocytogenes, Campylobacter jejuni*, and *Salmonella* spp. (Wang et al., 2013; Han et al., 2016; Komora et al., 2017). However, drug resistant strains of *Cronobacter* spp. were found in several studies (Lee et al., 2012; Xu et al., 2015; Fei et al., 2017), some of which were characterized as multidrug-resistant strains (Kilonzo-Nthenge et al., 2012). Therefore, it is necessary to investigate the antibiotic resistance of *Cronobacter* spp. recovered from various food samples in order to classify the patterns of resistance and to formulate an effective strategy to prevent the potential spread of these strains.

In recent years, attachment and biofilm formation of foodborne pathogens has become a matter of increasing concern for food safety research because the high likelihoods of potential cross-contamination may lead to serious food safety problems (Simoes et al., 2010). Recently, some researchers have found that strains of *Cronobacter* spp. were able to form biofilms on many kinds of materials such as stainless steel, polyvinyl chloride, silicone, and polycarbonate (Jo et al., 2010; Park and Kang, 2014). Established biofilms are very difficult to remove due to the tolerance to sanitizing agents, and thereby pose a potential health risk to human health because microorganisms within biofilms might result in a persistent release of bacteria to foods and environment. The aim of the present study was to investigate the genetic diversity, by MLST and serotyping, the antimicrobial susceptibility, and biofilm formation of 40 *Cronobacter* isolates from spices and cereals.

## Materials and methods

### Strain collection, culture condition, and DNA extraction

A total of 40 *Cronobacter* isolates recovered from spices and cereal food samples in China between September 2014 and June 2015 were analyzed (Table 1). Twenty-one strains were from spices and 19 from cereals. These strains have been confirmed as *Cronobacter* spp. by genus specific PCR confirmation based on the outer membrane protein A (*Omp*A) and internal transcribed spacer (ITS) gene, and 16S rRNA sequencing in our previous work (Li Y. H. et al., 2016; Li et al., 2017). The bacterial strains were routinely grown in Tryptic Soy Broth (TSB; QingDao Hope Bio-technology Co., Ltd, Qingdao, China) at 37°C overnight without shaking. Then genomic DNA was extracted with the EZNA Genomic DNA Isolation Kit (Omega Bio-Tek, Doraville, USA) according to the manufacturer's protocols.

**Table 1:** Molecular identification and biofilm formation profiles of *Cronobacter* strains used in this study.

**Origin**	**Strain**	**ID^a^**	***fus*A allele**	***fus*A sequencing**	**ST^b^**	**CC**	**Serotype^c^**	**Biofilm formation 595 nm**	**Biofilm formation category**
**SPICES**									
White pepper	XZCRO001	1705	**148**	*C. dublinensis*	**498**		NF	0.141 ± 0.012	Non-biofilm producer
White pepper	XZCRO002	1706	8	*C. sakazakii*	**495**		Csak O1	0.142 ± 0.004	Non-biofilm producer
White pepper	XZCRO003	1707	18	*C. sakazakii*	136		Csak O2	0.158 ± 0.012	Weak
White pepper	XZCRO004	1708	36	*C. sakazakii*	224		Csak O7	0.141 ± 0.018	Non-biofilm producer
Red pepper powder	XZCRO005	1709	20	*C. dublinensis*	**522**		Cdub O1	0.145 ± 0.014	Non-biofilm producer
Prickly ash powder	XZCRO006	1710	18	*C. sakazakii*	**500**		Csak O7	0.143 ± 0.017	Non-biofilm producer
Prickly ash powder	XZCRO007	1711	**149**	*C. sakazakii*	**501**		NF	0.174 ± 0.020	Weak
Prickly ash powder	XZCRO008	1712	26	*C. turicensis*	**502**		Ctur O3	0.129 ± 0.007	Non-biofilm producer
Dried bay leaves	XZCRO009	1713	22	*C. turicensis*	72	72	Ctur O3	0.132 ± 0.003	Non-biofilm producer
Chinese cinnamon	XZCRO010	1714	22	*C. turicensis*	72	72	Ctur O3	0.134 ± 0.007	Non-biofilm producer
Aniseed powder	XZCRO011	1715	7	*C. malonaticus*	**504**		Cmal O2	0.171 ± 0.020	Weak
Prickly ash powder	XZCRO012	1716	68	*C. sakazakii*	143	143	Csak O3	0.173 ± 0.010	Weak
White pepper	XZCRO013	1717	**144**	*C. dublinensis*	**570**		NF	0.202 ± 0.021	Weak
Fennel	XZCRO014	1718	**146**	*C. universalis*	**512**		Cuni O1	0.734 ± 0.034	Strong
Red pepper powder	XZCRO015	1719	**147**	*C. turicensis*	**506**		NF	0.142 ± 0.017	Non-biofilm producer
Red pepper powder	XZCRO039	1743	67	*C. sakazakii*	148	16	Csak O1	0.139 ± 0.010	Non-biofilm producer
Cumin	XZCRO040	1744	67	*C. sakazakii*	148	16	Csak O1	0.137 ± 0.015	Non-biofilm producer
Black pepper	XZCRO041	1745	13	*C. malonaticus*	**511**		Cmal O1	0.135 ± 0.017	Non-biofilm producer
Prickly ash powder	XZCRO042	1746	17	*C. sakazakii*	158		Csak O1	0.143 ± 0.012	Non-biofilm producer
**CEREALS**									
Mung bean flour	XZCRO016	1720	40	*C. malonaticus*	371		NF	0.145 ± 0.016	Non-biofilm producer
Red bean flour	XZCRO017	1721	20	*C. dublinensis*	**524**		Cdub O1	0.177 ± 0.030	Weak
Maize flour	XZCRO018	1722	40	*C. malonaticus*	371		NF	0.135 ± 0.003	Non-biofilm producer
Soybean flour	XZCRO019	1723	17	*C. sakazakii*	158		Csak O1	0.127 ± 0.004	Non-biofilm producer
Buckwheat flour	XZCRO020	1724	12	*C. sakazakii*	17	17	Csak O2	0.155 ± 0.006	Weak
Proso millet	XZCRO021	1725	36	*C. sakazakii*	224		Csak O7	0.126 ± 0.015	Non-biofilm producer
Black soya bean	XZCRO022	1726	67	*C. sakazakii*	148	16	Csak O1	0.123 ± 0.011	Non-biofilm producer
Wheat flour	XZCRO023	1727	67	*C. sakazakii*	148	16	Csak O1	0.175 ± 0.018	Weak
Buckwheat flour	XZCRO024	1728	7	*C. malonaticus*	129	129	Cmal O2	0.172 ± 0.009	Weak
Mung bean flour	XZCRO025	1729	20	*C. dublinensis*	175		NF	0.140 ± 0.009	Non-biofilm producer
Mung bean flour	XZCRO026	1730	1	*C. sakazakii*	4	4	Csak O2	0.146 ± 0.005	Non-biofilm producer
Glutinous rice	XZCRO027	1731	1	*C. sakazakii*	**508**		Csak O2	0.147 ± 0.015	Non-biofilm producer
Oatmeal flour	XZCRO028	1732	12	*C. sakazakii*	17	17	Csak O2	0.129 ± 0.009	Non-biofilm producer
Black rice	XZCRO029	1733	20	*C. dublinensis*	176		Cdub O1	0.136 ± 0.010	Non-biofilm producer
Maize flour	XZCRO030	1734	8	*C. sakazakii*	50		Csak O1	0.129 ± 0.012	Non-biofilm producer
Wheat flour	XZCRO031	1735	18	*C. sakazakii*	136		Csak O2	0.154 ± 0.008	Weak
Barley flour	XZCRO032	1736	8	*C. sakazakii*	68		Csak O2	0.159 ± 0.005	Weak
Maize flour	XZCRO033	1737	1	*C. sakazakii*	**509**		Csak O2	0.152 ± 0.003	Weak
Oatmeal flour	XZCRO034	1738	**146**	*C. universalis*	**510**		Cuni O1	0.160 ± 0.005	Weak
Wheat flour	XZCRO035	1739	14	*C. sakazakii*	13	13	Csak O2	0.146 ± 0.017	Non-biofilm producer
Soybean flour	XZCRO043	1747	67	*C. sakazakii*	148	16	Csak O1	0.381 ± 0.012	Moderate
Negative control								0.125 ± 0.008	

a *ID, Strain identification code in the Cronobacter PubMLST database*.

b *Newly determined alleles and STs are in bold type*.

c *NF, Not found*.

### Multilocus sequence typing and sequence analysis

MLST was performed by PCR amplification and sequencing of the fragments of typically 7 housekeeping genes (*atp*D, *fus*A, *gln*S, *glt*B, *gyr*B, *inf* B, and *pps*A) (Baldwin et al., 2009). Alleles and STs were assigned in accordance with the *Cronobacter* MLST database website (http://pubmlst.org/cronobacter/). The *fus*A allele sequence analysis was also performed with the aim to identify and differentiate the isolates into species as previously described (Alsonosi et al., 2015; Brandão et al., 2017).

### O-antigen serotype analysis

The serotypes of *Cronobacter* isolates obtained from spices and cereals in the present study were determined using the PCR-based O-antigen serotyping technique as previously described (Sun et al., 2012a,b; Jarvis et al., 2013). Primers and PCR cycling conditions used for serotyping of *Cronobacter* strains are listed in Table 2.

**Table 2:** Lists of primers and PCR cycling conditions used for serotyping of *Cronobacter* strains.

**Serogroup**	**Target gene**	**Primer sequence**	**PCR cycling conditions**	**Amplicon size (bp)**	**References**	**No. of strains**	
						**spices**	**cereals**
CsakO1	*wzy*	CCCGCTTGTATGGATGTT	95°C, 5 min; (94°C, 30 s; 53°C, 30 s; 72°C, 1 min) x 30; 72°C, 7 min	364	Sun et al., 2012b	4	5
		CTTTGGGAGCGTTAGGTT					
CsakO2	*wzy*	ATTGTTTGCGATGGTGAG	95°C, 5 min; (94°C, 30 s; 53°C, 30 s; 72°C, 1 min) x 30; 72°C, 7 min	152	Sun et al., 2012b	1	8
		AAAACAATCCAGCAGCAA					
CsakO3	*wzy*	CTCTGTTACTCTCCATAGTGTTC	95°C, 5 min; (94°C, 30 s; 53°C, 30 s; 72°C, 1 min) x 30; 72°C, 7 min	704	Sun et al., 2012b	0	1
		GATTAGACCACCATAGCCA					
CsakO4	*wzy*	ACTATGGTTTGGCTATACTCCT	95°C, 5 min; (94°C, 30 s; 53°C, 30 s; 72°C, 1 min) x 30; 72°C, 7 min	890	Sun et al., 2012b	0	0
		ATTCATATCCTGCGTGGC					
CsakO5	*wzy*	GATGATTTTGTAAGCGGTCT	95°C, 5 min; (94°C, 30 s; 53°C, 30 s; 72°C, 1 min) x 30; 72°C, 7 min	235	Sun et al., 2012b	0	0
		ACCTACTGGCATAGAGGATAA					
CsakO6	*wzy*	ATGGTGAAGGGAACGACT	95°C, 5 min; (94°C, 30 s; 53°C, 30 s; 72°C, 1 min) x 30; 72°C, 7 min	424	Sun et al., 2012b	0	0
		ATCCCCGTGCTATGAGAC					
CsakO7	*wzy*	CCCGCTTGTATGGATGTT	95°C, 5 min; (94°C, 30 s; 53°C, 30 s; 72°C, 1 min) x 30; 72°C, 7 min	364	Sun et al., 2012b	2	1
		CTTTGGGAGCGTTAGGTT					
CmalO1	*wzx*	AGGGGCACGGCTTAGTTCTGG	95°C, 2 min; (95°C, 30 s; 55°C, 30 s; 72°C, 1 min) x 25; 72°C, 5 min	323	Jarvis et al., 2011	1	0
		CCCGCTTGCCCTTCACCTAAC					
CmalO2	*wzx*	TGGCCCTTGTTAGCAAGACGTTTC	95°C, 2 min; (95°C, 30 s; 55°C, 30 s; 72°C, 1 min) x 25; 72°C, 5 min	394	Jarvis et al., 2011	1	1
		ATCCACATGCCGTCCTTCATCTGT					
CdubO1	*wzx*	TCGTTTTGATGCTCTCGCTGCG	95°C, 2 min; (95°C, 30 s; 55°C, 30 s; 72°C, 1 min) x 25; 72°C, 5 min	435	Jarvis et al., 2013	1	2
		ACAAATCGCGTGCTGGCTTGAA					
CdubO2	*wzx*	CTCGGTTCATGGATTTGCGGC	95°C, 2 min; (95°C, 30 s; 55°C, 30 s; 72°C, 1 min) x 25; 72°C, 5 min	227	Jarvis et al., 2013	0	0
		CAGCGTGAAAACAGCCAGGT					
CturO1	*wzx*	AGGGGCACGGCTTAGTTCTGG	95°C, 2 min; (95°C, 30 s; 55°C, 30 s; 72°C, 1 min) x 25; 72°C, 5 min	323	Jarvis et al., 2013	0	0
		CCCGCTTGCCCTTCACCTAAC					
CturO2	*wzy*	TTTCTTGTTATTGCCTGTGT	95°C, 5 min; (94°C, 30 s; 50°C, 30 s; 72°C, 1 min) x 30; 72°C, 5 min	438	Sun et al., 2012a	0	0
		AACAAAATCAGCGAGACTAA					
CturO3	*wzx*	GCATCCCTTCAGAGTAGCGCA	95°C, 2 min; (95°C, 30 s; 55°C, 30 s; 72°C, 1 min) x25, 72°C, 5 min	236	Jarvis et al., 2013	3	0
		ACCACCTGCCATTGTCCTACTG					
CuniO1	*wzx*	CATTCTCGCTTCCGCAGTTGC	95°C, 2 min; (95°C, 30 s; 55°C, 30 s; 72°C, 1 min) x25, 72°C, 5 min	145	Jarvis et al., 2013	1	1
		CCCAACCATCATTAGGGCCGAG					
Uncertain	–	–		–	–	4	3
Total						21	19

### Antimicrobial susceptibility testing

Antimicrobial susceptibility testing of *Cronobacter* strains was investigated by the Kirby-Bauer disk diffusion method using Mueller-Hinton agar (Hangzhou Microbial Reagent Co., Ltd, Hangzhou, China) according to the recommendations of the Clinical and Laboratory Standards Institute (CLSI, 2012). Thirteen antibiotics were tested: ampicillin (10 μg), ticarcillin-clavulanic acid (75:10 μg), cefixime (5 μg), amikacin (30 μg), gentamicin (10 μg), tetracycline (30 μg), ciprofloxacin (5 μg), nitrofurantoin (300 μg), chloramphenicol (30 μg), meropenem (10 μg), aztreonam (30 μg), ceftriaxone (30 μg), trimethoprim (5 μg). All *Cronobacter* isolates and the two quality control strains (*Escherichia coli* ATCC 29522 and *Staphylococcus aureus* ATCC 29213) were grown in nutrient agar plates (Hangzhou Microbial Reagent Co., Ltd, Hangzhou, China) at 37°C overnight during antimicrobial susceptibility testing.

### Biofilm formation assay

Microtiter plate assays (MPA) were performed to investigate the biofilm-forming ability of *Cronobacter* strains with minor modification, as previously described (Lee et al., 2012). Briefly, overnight cultures (1 ml) of *Cronobacter* strains (*n* = 40) were transferred to fresh TSB at 37°C for about 2 h in a shaking incubator. Subsequently, 200 μl of cell suspension (OD_600_ ≈ 0.3) was transferred into sterile 96-well flat bottom polystyrene microplates (Corning Inc., Corning, NY, USA). The plates were incubated statically at 37°C for 48 h. Then the microtiter plates were gently washed three times with 250 μl of sterile distilled water and dried at room temperature. The biofilm was stained with 200 μl of 0.1% crystal violet solution for 30 min and washed three times with 250 μl sterile water. After drying, the crystal violet was liberated by 200 μl of 95% ethanol following 10 min incubation at room temperature. Finally, the sterile TSB was used as negative control and the optical density (OD) value of each well was measured at 595 nm with a microplate reader (Bio-Tek Instruments, Winooski, VT, USA). All the experiments were performed three times.

The cutoff OD (ODc) was defined as three standard deviations (SD) above the mean OD of the negative controls. Based on the ODc, the *Cronobacter* isolates were classified into four categories: (1) non-biofilm producers: OD of test isolate ≤ ODc; (2) weak biofilm producers: ODc < OD of test isolate ≤ (2 × ODc); (3) moderate biofilm producers: (2 × ODc) < of test isolate ≤ (4 × ODc); (4) strong biofilm producers: OD of test isolate > (4 × ODc).

### Statistical analysis

Fisher's exact test was used to compare serotypes, antimicrobial susceptibility rates, or biofilm-formation abilities between *Cronobacter* isolates from spices and cereals. Statistical analysis was performed using the SPSS version 17.0 software package (SPSS Inc, Chicago, IL, USA). A *P*-value of < 0.05 was considered statistically significant.

## Results

### Species identification

**Figure 1:**
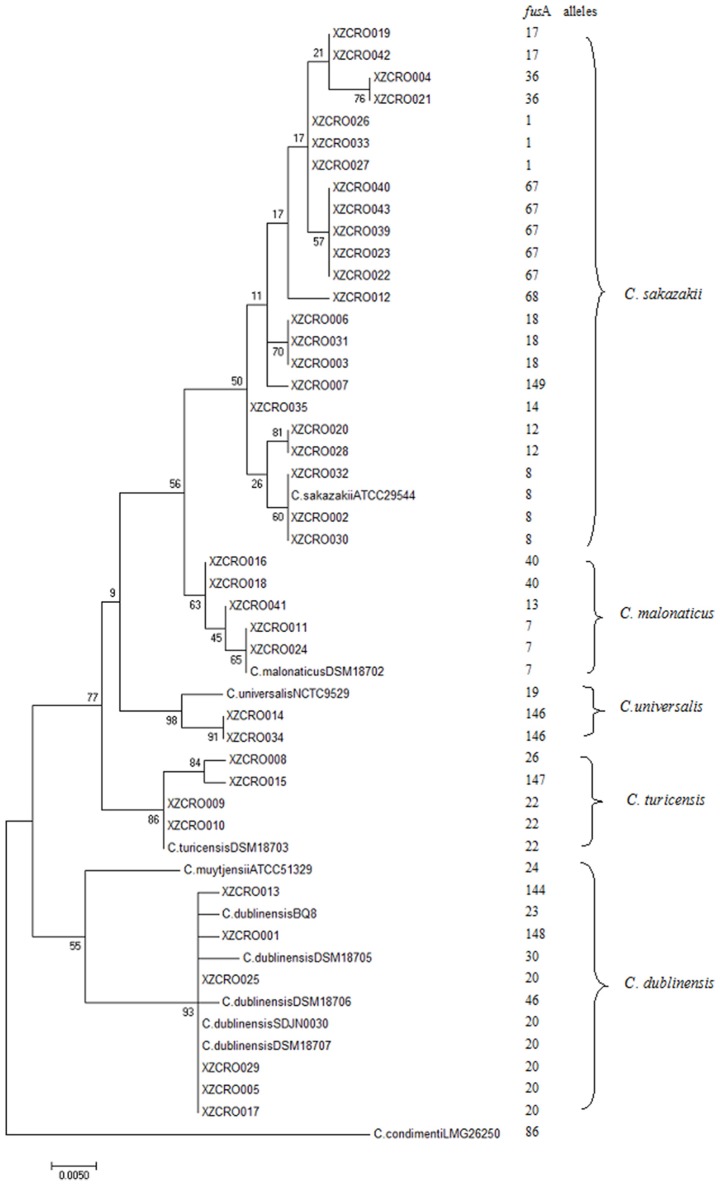
Maximum likelihood tree based on the *fus*A alleles (438 bp) for the differentiation of *Cronobacter* species in this study. This tree is drawn to scale using the ClustalX (V.2.0) and the MEGA (v.7.02) with 1,000 bootstrap replicates.

A total of 40 *Cronobacter* strains previously isolated from spices and cereal food samples were characterized by the *fus*A allele sequences analysis, and then all the allele sequences were submitted to the *Cronobacter* PubMLST database. A total of 21 *fus*A alleles (1, 7–8, 12–14, 17–18, 20, 22, 26, 36, 40, 67–68, 100, 144, and 146–149) were identified using the *Cronobacter* PubMLST database, four of which (146–149) were previously unreported (Table 1). Based on the *fus*A allele sequences analysis, a high diversity of *Cronobacter* species was observed, with five species of *Cronobacter* identified (Tables 1, 3). The most frequently observed isolates were *C. sakazakii* (*n* = 23), followed by *C. dublinensis* (*n* = 6), *C. malonaticus* (*n* = 5), *C. turicensis* (*n* = 4), and *C. universalis* (*n* = 2). No strains of *C. muytjensii* or *C. condimenti* were identified. The phylogenetic tree based on the *fus*A allele sequences demonstrates a very clear clustering across the genus *Cronobacter* with the 40 strains in five out of the seven species (Figure 1), which is in agreement with the results obtained from *fus*A allele sequences analysis.

**Table 3:** Summary of *fus*A alleles, MLST sequence types, and serotypes among different *Cronobacter* species.

**Bacterial species**	**No. of strains**	***fus*A alleles^a^**	**MLST sequence types^a^**	**Serotypes**
*C. sakazakii*	23	1, 8, 12, 14, 17, 18, 36, 67, 68, **149**	4, 13, 17, 50, 68, 134, 136, 143, 148, 158, 224, **495**, **500, 501, 508, 509**	CsakO1, CsakO2, CsakO3, CsakO7
*C. malonaticus*	5	7, 13, 22, 40	129, 371, **504, 511**,	CmalO1, CmalO2
*C. dublinensis*	6	20, 100, 144, **148**	175, 176, **498, 524, 570**	CdubO1
*C. turicensis*	4	22, 26, **147**	72, **502, 506**	CturO3
*C. universalis*	2	**146**	**510, 512**	CuniO1

a *New alleles and new STs are indicated in bold character*.

### Multilocus sequence typing

A total of 30 different STs among the 40 isolates were found, 14 (ST495, ST498, ST500-ST502, ST505, ST506-ST512, and ST570) of which were novel to the *Cronobacter* PubMLST database (Tables 1, 3). The most frequent STs in our study were ST148, identified five times, followed by ST17, ST72, ST136, ST158, ST224, ST371, and ST524 that included two isolates each, while the remaining 22 STs were identified only once. Of these frequent STs, the ST136, ST148, ST158, ST224, and ST524 were found in both spices and cereal samples; whereas ST17 and ST371 could only be found in cereal samples and ST72 found in spices samples. MLST assigned 12 isolates into 7 different CCs: CC4 (*n* = 1), CC13 (*n* = 1), CC16 (*n* = 1), CC17 (*n* = 1), CC72 (*n* = 1), CC129 (*n* = 1), and CC143 (*n* = 1), while the remaining 28 isolates were not assigned (Table 1).

### Serotyping by PCR

Of the 40 *Cronobacter* isolates, 33 (82.5%) were clearly identified by PCR-based O-antigen serotyping methods, while seven (17.5%) isolates were undefined since O-antigen gene could not be amplified. O-antigen serotyping classified these strains into 9 serotypes: *C. sakazakii* serotype O1 (*n* = 9), *C. sakazakii* serotype O2 (*n* = 9), *C. sakazakii* serotype O3 (*n* = 1), *C. sakazakii* serotype O7 (*n* = 3), *C. dublinensis* O1 (*n* = 3), *C. malonaticus* O1 (*n* = 1), *C. malonaticus* O2 (*n* = 2), *C. turicensis* O3 (*n* = 3), and *C. universalis* O1 (*n* = 2) (Tables 1, 2).

The serotype distribution of isolates from spices and cereals is shown in Table 2. A significant difference in the distribution of *Cronobacter* serotypes was observed between spices and cereals (*P* < 0.05). Analysis of the relationship between serotypes and MLST profiles revealed a connection between ST and serotype. For example, all strains genotyped as *C. sakazakii* ST158 were identified as *C. sakazakii* serotype O1, and *C. sakazakii* ST148 identified as *C. sakazakii* serotype O2. In contrast, isolates of the same serotype but different STs were found in this study. For example, isolates belonging to ST50, ST148, ST158, and ST495 were characterized as *C. sakazakii* serotype O1. Similarly, isolates belonging to ST4, ST13, ST17, ST68, ST136, and ST509 were characterized as *C. sakazakii* serotype O2.

### Antimicrobial susceptibility testing

All of the 40 *Cronobacter* isolates were susceptible to 10 of the 13 antibiotic agents tested including ampicillin, cefixime, amikacin, gentamicin, tetracycline, ciprofloxacin, nitrofurantoin, chloramphenicol, trimethoprim, and ticarcillin-clavulanic acid. However, 70.0% (28/40) of the strains were resistant to ceftriaxone, among which 27.5% (11/40) of the strains were found in spices and 42.5% (17/40) of the strains were found in cereals. Besides ceftriaxone, 25.0% (10/40) of the strains were resistant to meropenem, eight (XZCRO006:ST500, XZCRO007:ST501, XZCRO011:ST504, XZCRO012:ST143, XZCRO013:ST570, XZCRO014:ST512, XZCRO015:ST506, and XZCRO042:ST158) of which were detected in spices, while the remaining 2 isolates (XZCRO019:ST158, and XZCRO020:ST17) in cereals. In addition, 2 isolates (XZCRO009:ST72, and XZCRO040:ST148) from spices and only 1 isolate (XZCRO027:ST508) from cereals were resistant to aztreonam (Table 4). No multidrug resistance (isolates resistant to three or more antimicrobial agents) strains were observed in both spices and cereals. Majority of *Cronobacter* isolates with the same ST showed a similar drug-resistance profile. However, isolates with the same ST sometimes showed different drug-resistance profile. For example, the 5 strains (XZCRO022, XZCRO023, XZCRO039, XZCRO040, and XZCRO043) of *Cronobacter* belonged to ST148, but only one strain was resistant to aztreonam (XZCRO040:ST148). When susceptibility results were compared according to their sources, there was no significant difference in the prevalence of antimicrobial resistance between isolates from spices and cereals for any of the agents tested (*P* > 0.05).

**Table 4:** Antimicrobial susceptibility of the 40 *Cronobacter* strains recovered from spices and cereals by agar disc diffusion method.

**Antibiotic**	**No. of resistant strains (%)**		
	**Spices (*n* = 21)**	**Cereals (*n* = 19)**	**Total (*n* = 40)**
Ampicillin	0	0	0
Cefixime	0	0	0
Amikacin	0	0	0
Gentamicin	0	0	0
Tetracycline	0	0	0
Ciprofloxacin	0	0	0
Nitrofurantoin	0	0	0
Chloramphenicol	0	0	0
Trimethoprim	0	0	0
Ticarcillin-clavulanic acid	0	0	0
Aztreonam	2 (5.0)	1 (2.5)	3 (7.5)
Meropenem	8 (20.0)	2 (5.0)	10 (25.0)
Ceftriaxone	11 (27.5)	17 (42.5)	28 (70.0)

### Biofilm-formation ability of *Cronobacter* spp.

The biofilm-formation ability among the 40 isolates was detected by the MPA, and the results were shown in Table 1. Overall, a wide variation was found among the *Cronobacter* strains in the quantity of biofilm produced. The results indicated that 15 (37.5%) of the 40 tested isolates, belonging to 12 of the 30 previously identified STs, were capable to produce biofilm on polystyrene microtiter plates (Table 1). Using the proposed cutoff criteria, a cutoff value of 0.149 at OD_595_ nm was used to categorize the test strains as non-biofilm, weak, moderate, and strong biofilm producers. According to the result of microtiter plate test, one isolate belonging to ST512 scored as the most efficient biofilm producer, one isolate belonging to ST148 as moderate biofilm producer, and the other 13 isolates as weak biofilm producers (Table 1). However, no correlation between biofilm formation and STs was observed. *Cronobacter* strains identified as the same ST sometimes showed different biofilm-formation ability. For example, 5 strains (XZCRO22, XZCRO23, XZCRO39, XZCRO040, and XZCRO043) of *Cronobacter* were identified as ST148 in our study, only 1 of which (XZCRO043) was categorized as moderate biofilm producer, and 2 (XZCRO39 and XZCRO040) as weak biofilm producer, whereas the other 2 isolates (XZCRO22 and XZCRO23) were categorized as non-biofilm producers. In addition, there was no significant difference (*p* > 0.05) in the amount of biofilm detected for *Cronobacter* spp. between spices and cereals.

## Discussion

*Cronobacter* spp. have been isolated from many kinds of foodstuffs including plant materials such as vegetables, flours, herbs, and spices (Huang et al., 2015; Brandão et al., 2017), however the prevalence of *Cronobacter* spp. in such foodstuffs varied greatly among different studies. In a study of the prevalence of *Cronobacter* spp., these bacteria were detected in 26.7% (12/45) of herbs and spices in India (Singh et al., 2015). In another study, the prevalence of *Cronobacter* spp. was particularly low in spices samples (3.6%, 1/28) and dry cereals (4.9%, 6/123) in Netherlands (Kandhai et al., 2010). *Cronobacter* spp. was detected in herbs and spices, cereal mixes for children in Brazil (Brandão et al., 2017), where its prevalence was 36.7% (11/30) and 23.3% (7/30), respectively. In our previous studies, the overall prevalence of *Cronobacter* spp. in spices and cereals was determined to be 29.7% (19/64) (Li et al., 2017) and 21.0% (21/100) (Li Y. H. et al., 2016), respectively. However, in most of these studies, the MLST profiles of strains isolated from spices and cereals were not demonstrated. This study describes the genetic diversity, antimicrobial susceptibility, and biofilm formation of *Cronobacter* spp. recovered from spices and cereals in China during 2014–2015.

Based on the *fus*A sequence analysis, we found that the majority (57.5%) of *Cronobacter* isolates recovered from spices and cereals were *C. sakazakii*. The remaining strains were *C. dublinensis* (15.0%), *C. malonaticus* (12.5%), *C. turicensis* (20.0%), and *C. universalis* (5.0%). These findings are in agreement with previous studies which showed that *C. sakazakii* was the predominant *Cronobacter* species in different sources (Fei et al., 2015; Sulaiman et al., 2016; Brandão et al., 2017). Recent studies indicated that *C. sakazakii, C. malonaticus*, and *C. turicensis* were the three pathovars of *Cronobacter* spp. that associated with several neonatal infections and adult infections (Hunter and Bean, 2013; Ogrodzki and Forsythe, 2015). Unfortunately, these three pathovars of *Cronobacter* spp. were identified from spices and cereals in this study. These results underline the importance of sanitary-hygienic and epidemiological surveillance in spices and cereals to reduce the risk of *Cronobacter* infections.

The application of MLST analysis of *Cronobacter* isolates would be helpful to better understanding the genetic diversity, virulence, and epidemiology of genus *Cronobacter*. In this study, a total of 40 *Cronobacter* strains were genotyped with the 7-loci MLST scheme. MLST analysis revealed 16, 4, 5, 3, and 2 STs in *C. sakazakii, C. malonaticus, C. dublinensis, C. turicensis*, and *C. universalis*, respectively (Table 3). This finding was in agreement with previous studies reporting that the majority of STs were identified in *C. sakazakii* (Xu et al., 2015; Brandão et al., 2017). At the time of writing (August 2017), the *Cronobacter* PubMLST database contained 2097 isolates and consisted of 609 defined STs, with 225 clinical isolates belonging to 53 STs. The most frequent STs of clinical relevance in the *Cronobacter* PubMLST database were *C. sakazakii* ST4 (88/225), followed by *C. malonaticus* ST7 (30/225) and *C. sakazakii* ST8 (14/225). Among the 30 STs identified in our study, only 5 STs (ST4, ST13, ST50, ST129, and ST158) were of clinical origin, with 4 (ST4, ST13, ST50, and ST158) and 1 (ST129) ST(s) for *C. sakazakii*, and *C. malonaticus*, respectively. Among these 5 STs we identified, ST158, corresponding to *C. sakazakii*, was found in both spice (prickly ash powder) and cereal (soybean flour) samples, while ST4, ST13, ST50, and ST129 could only be found in cereal samples from mung bean flour, wheat flour, maize flour, and buckwheat flour, respectively. These findings underline that spices and cereals can also be potential sources of *Cronobacter* infections, which might pose great risks to human health.

Recent studies indicated a strong association between *C. sakazakii* CC4 (such as ST4, ST 15, ST97, and etc.) and neonatal infections as well as *C. malonaticus* CC7 (such as ST 7, ST 84, ST 159, and etc.) and adult infections (Joseph and Forsythe, 2011; Hariri et al., 2013; Forsythe et al., 2014). Moreover, a goeBURST analysis of 1007 *Cronobacter* isolates performed in 2014 indicated that 19.4% (*n* = 195) and 5.7% (*n* = 58) of strains in the *Cronobacter* PubMLST database were *C. sakazakii* CC4 and *C. malonaticus* CC7, with 45.1% (88/195) and 56.9% (33/58) strains obtained from clinical sources, respectively (Forsythe et al., 2014). These findings remark the importance of surveillance of *Cronobacter* belonging to *C. sakazakii* CC4 and *C. malonaticus* CC7, which are the dominant pathovars of *Cronobacter* associated with neonatal, pediatric and adult infections. However, these two CCs are not only found in powdered infant formula and related products but also in many other kinds of foodstuffs. For instance, in a study of the prevalence of *Cronobacter* contamination in 90 samples of retail foods in Brazil, two strains isolated from maize flour were characterized as *C. sakazakii* CC4 (Brandão et al., 2017). In another study, 4 *C. sakazakii* CC4 isolates were recovered from rice flour, noodle and potable water, and 10 *C. malonaticus* CC7 isolates from rice flour, dried shrimp, chocolate, cookie, and potable water (Cui et al., 2014). In our study, only one *C. sakazakii* CC4 isolate was obtained from cereals, and no strains of *C. malonaticus* CC7 were found in both cereals and spices.

For serotyping, a total of nine serotypes were found among the 40 isolates, including nine serotypes from spices and six from cereals. Among the nine serotypes found, *C. sakazakii* serotype O1 (*n* = 9) and O2 (*n* = 9) were the most two frequently observed serotypes, which was in accordance with previous studies (Alsonosi et al., 2015; Fei et al., 2015). Most *Cronobacter* isolates (*n* = 33) were clearly serotyped in this study, except for 3, 2, 1, and 1 isolate(s) in *C. dublinensis, C. malonaticus, C. sakazakii*, and *C. turicensis*, respectively. Previous studies also suggested that serotyping of *Cronobacter* strains were sometimes uncertain. For instance, 51 *Cronobacter* strains were isolated from hospitalized patients, one of which (identified as *C. muytjensii* ST28) could not be determined when the PCR serotyping scheme was carried out (Alsonosi et al., 2015). In another study, a total of 111 *Cronobacter* isolates from Chinese ready-to-eat foods were serotyped based on the O-antigen serotyping, two of which (one identified as *C. malonaticus* and the other as *C. dublinensis*) were uncertain (Xu et al., 2015). The appearance of unidentified serotypes may be due to the high genetic diversity of *Cronobacter* spp., which may result in a failure determination when the serotyping methods were performed in such studies. Recently, Ogrodzki and Forsythe established a new capsular typing scheme based on sequencing of *gnd* and *gal*E genes, which would be greatly helpful in distinguishing between *Cronobacter* species (Ogrodzki and Forsythe, 2015).

The increasing emergence of antibiotic resistant foodborne pathogens has been of great concern to public health in recent years. Results of the present study showed that frequency of antibiotic resistance in *Cronobacter* isolates recovered from spices and cereals was lower than strains of other foodborne pathogens such as *L. monocytogenes, C. jejuni*, and *Salmonella* spp. (Wang et al., 2013; Han et al., 2016; Komora et al., 2017). However, more attention should be paid to the inspection and control of strains of *Cronobacter* spp. because the resistance of these bacteria to many kinds of antimicrobial agents has been reported (Kilonzo-Nthenge et al., 2012; Li et al., 2014; Fei et al., 2017), even though the antimicrobial susceptibility profiles may vary in different studies performed in various samples collected from different locations.

Antimicrobial susceptibility testing revealed that the 40 isolates were susceptible to most antibiotics tested, except for ceftriaxone, meropenem, and aztreonam. Cephalosporins, the commonly used antimicrobial agents worldwide, were sometimes categorized into “generations” by their antimicrobial properties. The results of the present study suggested that a high resistance (70%) of *Cronobacter* spp. particularly *C. sakazakii* to ceftriaxone (third generation), whereas all isolates were sensitive to cefixime (third generation). Compared to our study, a little lower incidence (65%) of resistance to ceftriaxone was reported by Zhang et al. (2013) in imported dairy products; in contrast, antimicrobial resistance was not observed in another study performed by Li Z. et al. (2016) in retail milk-based infant and baby foods. Besides ceftriaxone, resistance of *Cronobacter* spp. to other cephalosporins, including cefazolin (first generation), cephalothin (first generation), and cefoxitin (second generation), has been reported in Iraq (Mossawi and Joubori, 2015) and UK (Gosney, 2008). The different performance of antimicrobial resistance on *Cronobacter* spp. among various cephalosporins might be due to extensive use or misuse of these antimicrobial agents which increased drug resistance of these bacteria. In our study, a total of 10 (25%) *Cronobacter* isolates were resistant to meropenem; in contrast, all of the tested isolates from dairy products including powdered infant formula in China, Iraq, and Japan were susceptible to meropenem (Oonaka et al., 2010; Pan et al., 2014; Li Z. et al., 2016). Apart from isolates originating from food, several clinical isolates were found susceptible to meropenem in Taiwan (Tsai et al., 2013).

In contrast to previous studies whereas resistance of *Cronobacter* spp. to ampicillin has been reported (Oonaka et al., 2010; Li et al., 2014; Fei et al., 2017), ampicillin-resistant strains were not found in this study. Besides ampicillin, *Cronobacter* isolates showed 100% susceptibility to tetracycline, ciprofloxacin and chloramphenicol, whereas the other researchers reported a high resistance of *Cronobacter* spp. to these antibiotics (Kilonzo-Nthenge et al., 2012). In one study conducted in the USA, high resistance of *C. sakazakii* isolated from domestic kitchens to tetracycline (66.6% of isolates) and ciprofloxacin (57.1%) was observed. In another study in South Korea, Lee et al. (2012) reported that 3.4 and 1.8% of *Cronobacter* isolates recovered from various types of foods were resistant to chloramphenicol and tetracycline, respectively.

In the present study, 37.5% of the *Cronobacter* isolates from spices and cereals were able to form biofilm on polystyrene surfaces; however majority of these isolates (32.5%) were weak biofilm producers and less were moderate (2.5%) or strong (2.5%) biofilm producers. Similar results have been reported earlier in Mexico wherein 26% of *Cronobacter* spp. was capable of forming biofilms (Cruz et al., 2011). In contrast, a high proportion of biofilm-producing isolates of *Cronobacter* spp. recovered from various food in South Korea was observed (Lee et al., 2012). Differences in biofilm formation between various *Cronobacter* isolates could be due to strain variations that recovered from different sources and geographical locations. Moreover, the capacity of biofilm formation of *Cronobacter* strains is generally influenced by environmental conditions such as culture media and carbon source, and storage humidity levels (Jung et al., 2013).

## Conclusion

In conclusion, the present study demonstrated a high genetic diversity of *Cronobacter* isolates recovered from spices and cereals, providing useful information on molecular epidemiology of *Cronobacter* infections. MLST analysis revealed that *C. sakazaki* was the most common species recovered from spices and cereals, followed by *C. dublinensis C. malonaticus, C. turicensis*, and *C. universalis*. The presence of isolates of clinical relevance including *C. sakazakii* ST4 (CC4) revealed that spices and cereals are likely to be the potential sources for human infection with *Cronobacter* spp. Although most *Cronobacter* strains were susceptible to the antimicrobial agents used in this study, further studies on the antimicrobial resistance of these foodborne pathogens are important to ensure effective treatment of human infections caused by *Cronobacter* spp.

## Author contributions

YL and JS: Contributed to the conception of the study; YL and HY: Wrote the manuscript; YL and YZ: Analyzed and interpreted the data; YL, HJ, and YJ: Conducted the experiments; Each author substantially contributed to the work reported here.

### Conflict of interest statement

The authors declare that the research was conducted in the absence of any commercial or financial relationships that could be construed as a potential conflict of interest.
